# Treatment with Growth Hormone for Adults with Growth Hormone Deficiency Syndrome: Benefits and Risks

**DOI:** 10.3390/ijms19030893

**Published:** 2018-03-17

**Authors:** Juan J. Díez, Susana Sangiao-Alvarellos, Fernando Cordido

**Affiliations:** 1 Department of Endocrinology, Hospital Ramón y Cajal, 28034 Madrid, Spain; juanjose.diez@salud.madrid.org; 2 Department of Medicine, University of Alcalá de Henares, 28034 Madrid, Spain; 3 Faculty of Health Sciences, University of A Coruña, 15006 A Coruña, Spain; Susana.Sangiao.Alvarellos@sergas.es; 4 Instituto de Investigación Biomedica (INIBIC), University Hospital A Coruña, 15006 A Coruña, Spain; 5 Department of Endocrinology, University Hospital A Coruña, Xubias de Arriba 84, 15006 A Coruña, Spain

**Keywords:** growth hormone, growth hormone deficiency, hypopituitarism, treatment

## Abstract

Pharmacological treatment of growth hormone deficiency (GHD) in adults began in clinical practice more than 20 years ago. Since then, a great volume of experience has been accumulated on its effects on the symptoms and biochemical alterations that characterize this hormonal deficiency. The effects on body composition, muscle mass and strength, exercise capacity, glucose and lipid profile, bone metabolism, and quality of life have been fully demonstrated. The advance of knowledge has also taken place in the biological and molecular aspects of the action of this hormone in patients who have completed longitudinal growth. In recent years, several epidemiological studies have reported interesting information about the long-term effects of GH replacement therapy in regard to the possible induction of neoplasms and the potential development of diabetes. In addition, GH hormone receptor polymorphism could potentially influence GH therapy. Long-acting GH are under development to create a more convenient GH dosing profile, while retaining the excellent safety, efficacy, and tolerability of daily GH. In this article we compile the most recent data of GH replacement therapy in adults, as well as the molecular aspects that may condition a different sensitivity to this treatment.

## 1. Introduction

The syndrome of growth hormone deficiency (GHD) in adulthood has been fully defined [1,2] and is characterized by alterations in body composition, decreased capacity for exercise and quality of life (QoL), as well as a series of unfavorable changes in cardiovascular function, and lipid and carbohydrate metabolism. Its diagnosis is based on the combination of pituitary disease, hypopituitarism and a decrease in the concentration of insulin-like growth factor I (IGF-I) or in diminished GH responses to different stimuli. A concentration of IGF-I below normal for age and sex in a patient with involvement of three or more pituitary axes is diagnostic of GHD. Expert consensus defines severe GHD as a GH response peak after insulin hypoglycemia below 3 μg/L. This is an arbitrary limit that depends on the assay method, the laboratory, and the type of stimulation. Other stimuli used have been the GH-releasing hormone (GHRH) plus arginine, GHRH plus GH-releasing peptide (GHRP-6), glucagon, or the long oral glucose test [3,4,5].

Replacement therapy with recombinant human GH has been available since the 1980s. The experience accumulated since then is extensive, and nowadays there is no doubt that this therapy improves or reverses most of the signs and symptoms of this hormonal deficiency (Table 1). However, chronic GH administration is not without potential risks (Table 1).

We herein review the most recent data and summarize our current knowledge about the benefits and risks of GH replacement therapy in adults deficient in this hormone. All articles related to replacement therapy of growth hormone deficiency were searched in MEDLINE from 1985 to 2017. The search was performed by using the terms “replacement therapy” and “adult” as subheadings of the term “growth hormone deficiency” in the Medical Subject Headings (MeSH) thesaurus. All clinical trials and systematic reviews related to growth hormone deficiency were also searched at the Cochrane Database of systematic Reviews and The Cochrane Central Register of Controlled Trials (CENTRAL). The selection criteria included all prospective and retrospective studies, all case series, all case reports, and reviews concerning the effects of growth hormone replacement therapy on adult patients with GHD. Duplicated articles were excluded. This search was supplemented by a review of reference lists of potentially eligible studies and a manual search of key journals in the field of endocrinology.

## 2. Benefits of Treatment with Growth Hormone

GH replacement therapy is associated with beneficial effects on body composition, bone structure, health-related QoL and several cardiovascular risk factors [6,7].

### 2.1. Body Composition

Initial studies showed that treatment with GH induced a decrease in fat mass and an increase in lean mass [8]. Elbornsson et al. [9] in a cohort of 156 patients with GHD, reported an improvement in lean mass maintained for 15 years, and a marked initial decrease in fat mass, followed by a slowly progressive increase over time, in possible relation with the aging process. A recent systematic review concluded that the long-term effects on body mass index appear to be inconclusive, with some studies reporting an increase and other reporting no change [7]. On the other hand, most long-term studies report no effect of GH replacement therapy on waist-hip ratio or waist circumference. A slight but significant increase in waist circumference has been reported in one study [10]. It has been postulated that the observed increase in body mass index and waist circumference in some studies is in line with changes due to normal aging process [11,12]. In other words, it is possible that in long-term studies the aging process attenuates some of the beneficial effects of GH treatment on body composition.

Interestingly, data from the study by Filipsson Nystrom et al. [13] showed that discontinuation of GH therapy for four months, after more than three years of treatment, was followed by an increase in abdominal subcutaneous and visceral fat and a decrease in thigh muscle mass. A recent meta-analysis of 22 trials, including 591 GH- and 562 placebo-treated patients, found that mean lean body mass increased by 2.61 kg in GH-treated subjects versus 0.04 in the placebo group, and that fat mass was reduced by 2.19 kg versus 0.31 (GH vs. placebo). Changes in lean body mass and fat mass were dose-related, with high dose (HD) being more effective than low dose [14]. 

Lean body mass, including both skeletal muscle mass and tissue hydration, is increased with GH replacement therapy [15]. GH therapy causes an increase in the tubular reabsorption of sodium in the distal nephron. This is accompanied by an increase in plasma renin activity and decreased brain natriuretic peptide levels [16]. In addition, although treatment with GH is accompanied by an increase in lean body mass, most studies do not allow differentiating between extracellular water and intracellular mass [7]. Therefore, the lean body mass data may not be accurate, as GH replacement is associated with an increase in the intracellular water component. However, GH replacement therapy increases muscle strength and exercise capacity in patients with GH deficiency. In the study by Gotherstrom, GH treatment during 10 years in patients with GHD increased muscle strength during the first half of the study and later protected from the decline in muscular strength that occurs with aging [17]. These data, although indirectly, clearly suggest a real increase in muscle mass.

### 2.2. Bone Structure

Replacement therapy with GH in adult patients with GHD increases bone mineral density (BMD) and helps to optimize peak bone acquisition in patients who have persistent GH deficiency during the transition from adolescence to adulthood [6,7,18].

The effect on BMD is greater at vertebral than femoral level and after 18–24 months of treatment, and most studies show an increase of 4–10% of BMD [19]. In patients with childhood-onset GHD it has been shown that the continuation or reinstitution of treatment for two years, in patients who completed growth, induced a significant increase in BMD compared to untreated patients [20]. Therefore, the continuation of GH treatment during the period of transition from childhood to adulthood is recommended to obtain complete bone maturation. A prospective study in 18 patients with GHD showed that seven years of GH treatment induced an increase in lumbar spine BMD, which stabilizes during long-term therapy. However, a significant positive effect on bone microarchitecture could not be demonstrated in these patients [21]. Furthermore, two recent meta-analyses have suggested that the beneficial effect of GH therapy on BMD in adults with GHD is mainly affected by gender, age, dose and treatment duration [22,23]. In a cohort of 230 adult GHD patients, followed up to 15 years, Appelman-Dijkstra et al. [24] demonstrated a sustained increase in BMD at the lumbar spine, particularly in men, and stabilization of BMD values at the femoral neck. This study suggested that the clinical fractures incidence was no increased during long-term GH replacement [24].

Estrogenic hormonal status exerts also influence on the GH replacement therapy effects on bone metabolism. In a prospective, single-center study, including 87 consecutive patients (52 men and 35 women) with adulthood onset GHD, GH replacement induced a sustained increase in total, lumbar and femoral neck BMD and bone mineral content. There was a tendency for women on estrogen treatment to have a greater increase in bone mass and density compared with women without estrogen replacement. This study suggests that adequate estrogen replacement is needed in order to have an optimal response in bone mineral density in GHD women [25].

### 2.3. Quality of Life

GH treatment improves health-related QoL in the majority of patients. Most of the improvement in QoL occurs during the first year of treatment, although this beneficial effect persists in the medium and long term [26]. Sustained improvement in QoL scores has been shown to be more marked in women and in patients with low QoL at baseline [27,28].

A special group consists of patients with a previous history of acromegaly who subsequently presents GHD. This particular group of patients exhibits a significant deterioration of health-related QoL that is improved by GH replacement [29].

### 2.4. Cardiovascular Risk Markers

Treatment with GH in patients with GHD improves several cardiovascular risk factors, such as lipid profile, endothelial function and cardiovascular inflammatory markers [30,31].

Dyslipidemia has been considered the strongest contributor of the excess in cardiovascular risk associated with hypopituitarism [31]. Most studies have shown an increase in high-density lipoprotein (HDL) cholesterol and a decrease in total cholesterol and low-density lipoprotein (LDL) cholesterol after administration of GH [32]. Withdrawal of GH treatment for four months, after more than three years of administration, was accompanied by an increase in total and LDL cholesterol [13], which indirectly shows one of the great benefits of the GH therapy in patients with GHD. The positive effect of GH therapy on lipid profile has been confirmed in a long-term 15-year prospective study [9]. Furthermore, a slight decrease in diastolic blood pressure has been demonstrated in a meta-analysis of placebo-controlled studies [33]. Inflammatory markers are elevated in patients with GHD and treatment with GH can improve low-grade inflammation, as documented by a reduction in C-reactive protein, TNF-α, and interleukin-6 [13,31,34].

Increased thickness of the carotid intima-media is an important predictor of coronary disease in epidemiological studies. Replacement with GH in patients with GHD has been shown to decrease this parameter [35]. The study by Makimura et al. [36] is of particular interest in this context. In patients with obesity and decreased GH secretion, randomized and placebo-controlled administration of a GHRH analogue induced a decrease in visceral fat mass and in the intima-media thickness of the carotid, in addition to a moderate elevation of IGF-I [36].

### 2.5. Influence of Severity

Patients who are most likely to benefit from GH replacement therapy include those with severe GH deficiency, defined by the response to GH provocative testing and serum IGF-1 levels, including patients with genetic and other congenital causes of GH deficiency. Retesting during the transition to adulthood in children with idiopathic GH deficiency is necessary but is not mandatory in patients expected to have lifelong severe GH deficiency owing to causes such as genetic mutations, structural lesions, or deficiencies of multiple pituitary hormones. In addition, continued GH replacement therapy during the transition period after completion of linear growth is recommended to permit achievement of full skeletal and muscle maturation [15].

### 2.6. Influence of Age

The patient’s age should be taken into account when considering GH replacement therapy. GH secretion normally decreases with age, and older patients have an increased susceptibility to GH-related side effects. Therefore, GH dose requirements are lower in older patients and higher in some transition and young adult patients [20], especially if serum IGF-I are very low [37]. On the other hand, there are no well-conducted prospective studies on the effect of age on the benefits and risks of GH replacement. In a systematic review of eleven studies in patients older than 60, treatment with GH decreased total and low density lipoprotein cholesterol levels, but did not alter high density lipoprotein or triglyceride levels. In addition, GH did not affect body mass index, blood pressure, or bone mineral density but decreased waist circumference, increased lean body mass, and decreased total fat mass. GH replacement consistently improved QoL.

There are no data on the efficacy and safety of GH treatment in patients older than eighty years [38]. In a prospective, single-center, open-label study, the effects of GH replacement were determined in 24 GHD adults above 65 years of age and in 24 younger GHD patients. Despite the lower dose in the elderly group, these patients had a more marked reduction in waist/hip ratio and serum low-density lipoprotein-cholesterol level, and these differences remained also after correction for duration of hypopituitarism. There was no difference at baseline or in responsiveness in lean mass, bone mineral density, and glucose homeostasis [39].

### 2.7. Other Beneficial Effects

Sleep disturbances have been reported in patients with GHD. Morselli et al. [40] have shown that four months of GH replacement therapy partly reversed sleep disturbances previously observed in untreated patients [40]. Cardiac size and cardiac performance have been reported to improve with GH replacement therapy [34]. A prospective study in 14 patients with adult-onset GHD showed that one year of GH therapy improves coronary flow reserve and left ventricular diastolic function in the patient population analyzed, thereby encouraging the early start of GH replacement therapy [41]. Six months of GH treatment significantly improved anaerobic capacity and physical function in a time-dependent manner in adults with GHD [42].

Retrospective [43,44] and prospective [45] studies have shown that patients with hypopituitarism treated with usual replacement therapy without GH exhibit an increased mortality in comparison with general population, especially concerning CV disease. In a group of 1411 hypopituitary adults without GH replacement, Svensson et al. [44] found that overall mortality and the rates of myocardial infarctions, cerebrovascular events, and malignancies were increased compared with the normal population. In a cohort of 289 patients on GH replacement, overall mortality and the rate of malignancies were similar to the normal population. The rate of myocardial infarctions was lower than that in the background population, although there was a tendency toward an increased rate of cerebrovascular events in this cohort. Therefore, it has been suggested that GH replacement appeared to provide protection from myocardial infarctions [44].

## 3. Risks of GH Replacement Therapy

### 3.1. Body Composition

Not all GH-induced changes in body composition are favorable. The waist circumference and the body mass index (BMI) increase significantly in some studies [46]. A systematic review of 23 prospective studies of GH treatment for 5–15 years showed an increase in BMI, waist circumference and waist-hip index. In addition, although treatment with GH is accompanied by an increase in lean body mass, most studies do not allow differentiating between extracellular water and intracellular mass [7].

### 3.2. Bone Structure

Childhood-onset GHD is associated with a reduction in bone mass, but this is not the case in adult-onset GHD [47,48]. Best responses are seen in men and in patients with lower BMD at the beginning of treatment. However, studies do not detail the use of calcium, vitamin D or antiresorptive drugs [7], being difficult to draw conclusions. In addition, to date, no randomized controlled study has shown effects of GH treatment on the rate of fractures.

### 3.3. Quality of Life

A recent retrospective analysis of patients who were treated with GH during childhood in 11 centers of pediatric endocrinology in Switzerland compiled the information on QoL during adult life. Data of 300 participants showed that QoL results are in relation with the underlying indication for GH treatment. In particular, patients with associated diseases or syndromes scored slightly lower, and former cancer patients scored lower than patients with isolated GHD or idiopathic short stature. Lower physical component summary was associated with lower educational level [49]. These data emphasize the need to bear in mind the underlying condition when interpreting results of QoL measurements in adults with childhood onset GHD.

QoL improvements have not been reported in all adults or in all assessed socio-psychological domains [50,51]. Some studies have shown even worsening in some domains, such as social function and mental health, in adults with childhood-onset GHD [52]. In general, responses are very heterogeneous and patients with worse QoL before treatment are the best responders [27,28]. Therefore, it is possible that patients who have a normal QoL at baseline do not benefit from GH treatment in this regard.

### 3.4. Cardiovascular Risk Markers

Some authors have shown that lipoprotein (a), an independent marker of cardiovascular risk, increases significantly during treatment with GH [53]. In addition, one study has observed an increase in atherosclerosis plaques after six months of GH replacement therapy in adults with previously untreated congenital GHD due to mutation of the GHRH receptor [54].

### 3.5. Adverse Events

Side effects derived from water retention caused by GH occur in 5–18% of patients. They are edema, arthralgias, myalgias, paresthesias, and carpal tunnel syndrome, and usually improve after dose reduction. Elderly, female, and overweight patients have a higher risk of developing these effects. Retinopathy and benign intracranial hypertension are very rare complications of GH treatment. The needs of cortisol and thyroxin may be increased in patients with hypopituitarism [55]. Special attention should be given to older, heavier, and female patients with GHD because they are more susceptible to adverse events. Monitoring IGF-I concentrations is essential to avoid adverse effects of excess GH treatment. A recent randomized, open-label, clinical trial including 32 adults receiving GH therapy for at least one year showed that although increasing GH dose to achieve IGF-I levels between 1 and 2 Standard Deviation Score (SDS) improved waist circumference and the overall better feeling, safety was not guaranteed with the demonstrated effect on HDL cholesterol in men, and reported myalgia [56].

To minimize the impact of adverse effects, it is appropriate to start treatment with low doses and titrate upwards according to clinical response and IGF-I levels, as well as following some general recommendations that are shown in Table 2.

### 3.6. Risk of Hyperglycemia and Diabetes

Treatment with GH reduces insulin sensitivity and elevates blood glucose. In one study, conducted in 90 patients on GH treatment, a significant increase in both glucose and hemoglobin A1c levels was demonstrated. Changes were evident at six months and persisted after two years of treatment [57]. These data have been corroborated in some studies [58] and by a meta-analysis of 37 randomized, placebo-controlled, clinical trials, which showed that GH treatment was accompanied by an increase in fasting blood glucose and insulin concentrations, regardless of the dose and duration of treatment [33]. In a randomized, double-blind, placebo-controlled clinical trial in 166 adult patients with GHD, treatment with GH was associated with a worsening of glucose tolerance with the appearance of diabetes in 4% and carbohydrate intolerance in 20% of patients after 12 months, while in the placebo group only 8% developed intolerance [32].

In the Hypopituitary Control and Complications Study (HypoCCS) the prevalence and incidence of diabetes in adults treated with GH has also been analyzed [59]. Results of an analysis of 2922 patients in the United States and 3709 in Europe, with a mean follow-up of 4.1 years, showed that, in the United States, the incidence rate of diabetes adjusted for age, sex, and BMI is higher in patients treated with GH than in the general population. In France and Germany, the incidence rates were comparable with the reference population, while in Sweden the incidence rate was more than double in patients on GH treatment. A more recent analysis of 5143 patients from the Pfizer International Metabolic Database (KIMS), with 20,106 patient-year follow-up showed that, in southern Sweden, the observed/expected cases ratio was 10.8 the first year of treatment with GH and fell to 1.9 after eight years of treatment, but always stayed above 1. When the incidence of diabetes in patients in the KIMS study was compared with the incidence rates in age-adjusted populations in other European and US regions, the observed/expected cases ratios ranged from 2.11 to 5.22 [60].

The Safety and Appropriateness of Growth Hormone Treatment in Europe (SAGhE) was a population-based cohort study that analyzed the long-term mortality and morbidity in adults who were treated with GH during childhood. A recent report by Poidvin et al. [61] have used the French SAGhE database to evaluate the prevalence of diabetes, including gestational diabetes, in a population-based cohort of patients treated with GH for short stature during childhood, and to compare this prevalence with that of the general population in France. They analyzed a cohort of 5100 children with idiopathic short stature, or short stature in children born short for gestational age. In summary, these authors found no increase in the risk of treated diabetes in subjects receiving GH treatment during childhood, with a mean follow-up of 19 years.

A recent study in 245 patients with adult-onset GHD and more than four years of GH replacement showed that this therapy did no adversely affect glucose homeostasis in the majority of adults patients, although seven patients developed diabetes [62]. A meta-analysis of 94 randomized controlled trials and open trials did not demonstrate neither an increased frequency of diabetes in the short-term placebo controlled trials or a consistently increased incidence of diabetes during long-term GH replacement. However, the small number of subjects and the absence of adequate control population are a limitation for the interpretation of these results [63].

Close monitoring of glucose status is advisable during GH treatment in patients with obesity or a family history of type 2 diabetes, because they are more prone to develop impaired glucose intolerance and diabetes during therapy [64].

### 3.7. Risk of Neoplasia

Epidemiological studies have suggested an association of high circulating IGF-I or GH to cancer incidence [65,66], and the incidence of some malignant neoplasia is known to be increased in acromegaly [67]. Initial studies conducted in children treated with extractive GH showed an increased risk of mortality from cancer and, in particular, from colorectal cancer and Hodgkin’s disease [68]. The oncogenic risk during treatment in children treated with GH was established in the National Cooperative Growth Study (NGCS) and the Pfizer International Growth Database (KIGS), which evaluated more than 50,000 patients each, with almost 200,000 patient-years each. The standardized incidence rate of cancer was 1.12 (95% confidence interval: 0.75–1.61) in the former [69] and 1.26 (0.86–1.78) in the latter [70], thus suggesting that there was no significant increase in the risk of cancer in these children. No greater risk of leukemia than the general population could be demonstrated in these studies. Furthermore, recent studies have not confirmed an increased risk of mortality in children treated with GH, especially when applying sex-specific mortality models adjusting for birth characteristics [71].

The risk of second neoplasia in survivors of childhood cancer has been analyzed in depth in the Childhood Cancer Survivor Study (CCSS). In a scrutiny conducted in 2002, this study showed that children treated with GH had a relative risk for cancer of 3.21 (1.88–5.46) in comparison to cancer survivors not treated with this hormone [72]. This relative risk was even higher, 4.98 (1.95–12.74), in leukemia survivors. In the 2006 analysis [73], including a further 32-month follow-up, the risk of second neoplasia was reduced to 2.15 (1.33–3.47), but remained significant. Another study in one center, including 49 patients with GHD within a cohort of 310 childhood cancer survivors, showed that GH replacement therapy did not seem to increase the risk of second neoplasia, but the authors suggest the need of a close long-term follow-up in these patients [74].

Data available in adult patients suggest that replacement therapy with GH does not increase the rate of recurrence or progression of tumors in the hypothalamic-pituitary area [75]. 

An analysis of the risk of neoplasia was carried out in the SAGhE study database, including 6928 patients treated for idiopathic GHD, neurosecretory dysfunction, idiopathic short stature and small for gestational age, that is, etiologies of GHD without increased risk of neoplasia. Mean time of follow-up after completing therapy was 7.8 years [76]. In this study, the standardized mortality ratio for bone and cartilage neoplasia reached the striking figure of 5.00 (1.01–14.61). In a group of 6840 adults included in the HypoCCS study, the incidence ratio of neoplasia was 0.88 (0.74–1.04), but it rose to 3.79 (1.39–8.26) when patients younger than 35 years were analyzed, and to 2.74 (1.18–5.42) when analyzing patients with childhood onset GHD [77]. These results suggested that the overall risk of primary cancer in adult life is not increased; however, there are subgroups with high risk.

Using the HypoCCS database, Child et al. [78] reported the incidence of primary neoplasia in a cohort of 8418 patients treated with GH, as well as in 3668 GH-treated patients with history of pituitary adenoma and 956 GH-treated patients with history of craniopharyngiomas. Comparison were carried out with cohorts of untreated patients. During a mean follow-up of 4.8 years, they found that no increased risk for all-site cancers, including breast, prostate and colorectal cancers, in GH-treated patients. In addition, GH treatment did not increase the risk of recurrence of pituitary adenoma or craniopharyngioma in this study.

Recent studies have also reported reassuring results. A recent study, including 426 patients with nonfunctioning pituitary adenomas, with 4599 patient-years of follow-up, of whom 207 had used GH therapy and 219 had not received GH, showed a reduced overall mortality in GH-treated patients compared with the general population and found that death due to malignancy was not increased in GH-treated patients [79]. However, selection bias explaining some of the results cannot be excluded in this study. On the other hand, a recent metaanalysis, including two retrospective and seven prospective studies with a total of 11,191 participants, suggested that GH replacement therapy could reduce risk of cancer in adult with GHD [80].

Nevertheless, GH therapy should not be given to patients with active malignancy [64], and should be prescribed with caution in GHD adult patients with a history of cancer, strong family history of cancer, and advancing age [81,82]. Childhood cancer survivors may be at increased risk for secondary neoplasm compared with general population. GH should be used cautiously in this subgroup of patients [81].

### 3.8. Mortality Risk

A study conducted with data from the Dutch National Registry of GH treatment [83] compared 2229 patients undergoing treatment with GH with a primary control group of 109 patients diagnosed but not treated with GH and a secondary control group of 356 patients treated with GH in whom the treatment had been discontinued. The standardized mortality ratio in relation to the general population was 1.27 (1.04–1.56) for the treatment group. This ratio was 1.29 (1.05–1.59) when patients with acromegaly and Cushing’s disease were excluded, and 1.00 (0.79–1.26) after the exclusion of high-risk patients (craniopharyngioma and other causes). It is noteworthy that the authors found no significant increase in mortality in the two untreated control groups. The authors also found a significant increase in the standardized mortality ratio due to cardiovascular disease in women in the treatment group (2.52 [1.57–4.06]), which persisted after exclusion of high-risk patients.

The evaluation of a group of 13,983 GH-treated patients, with a mean follow-up of 4.9 years, showed that all-cause mortality in these patients was 13% higher than in the general population (standardized mortality ratio of 1.13 [1.04–1.24]). There was no increase in mortality due to cardiovascular disease or cancer [84]. In the aforementioned SAGhE study, the risk of overall mortality in patients who received GH in childhood increased by 33%. In this study, the standardized mortality ratio for circulatory diseases was 3.07 (1.40–5.83), and rose to 5.29 (1.42–13.55) and 6.66 (1.79–17.05) when deaths due to cerebrovascular disease and intracranial hemorrhage were considered, respectively. On the contrary, the observational GeNeSIS study, evaluating 9504 GH-treated children followed for at least four years found no increase in risk of mortality compared with children in the general population [85]. This study found an increase in mortality risk for children with history of malignant neoplasia. However, this study is limited to the period of active treatment with GH and without follow-up in adult life.

In summary, as stated in the Endocrine Society clinical practice guideline [6], although mortality is increased in patients with hypopituitarism and GHD has been implicated in this, there is no data to show that treatment with GH improves the survival of patients.

## 4. Cellular and Molecular Conditionings of GH Therapy

GH initiates it action by binding to growth hormone receptors (GHR) present on the plasma membrane of target cells. GHRs belong to the class I cytokine receptor family along with prolactin, erythropoietin, leptin and interleukins receptor [86,87]. The GHR receptor polymorphism has been extensively studied [88]. The GHR is a membrane receptor composed of 638 amino acids with three cellular domains: an extracellular, a transmembrane and an intracellular domain. The extracellular domain attaches to the circulating GH and corresponds to the circulating GH binding protein [89]. Activation of the receptor is induced through the interaction of GH to a preformed GHR dimer, leading to a conformational change in the intracellular domain that results in the phosphorylation and activation of STAT5 through Janus Kinase 2 [90,91,92]. 

The human GHR gene is located in chromosome 5, the mouse GHR gene is located on chromosome 15 [93,94]. In the human gene there are nine exons that encode the receptor and several additional exons in the 5′ untranslated region. The coding exons span at least 87 kilobase pairs of chromosome 5. There are two major isoforms of the GHR that differ by the absence of exon 3 that encodes part of the extracellular domain of the GHR. Its absence gives rise to a GHR lacking 22 amino acids in the extracellular domain [93,94]. The first studies of GHR with variations in the coding region came from the work of Godowski et al. [94] on Laron syndrome, an autosomal recessive genetic disorder that is characterized by resistance to GH. The isoform of human GHR containing exon 3 is known as the full-length isoform (fl-GHR) and the isoform without exon 3 as the exon 3-deficient isoform (d3-GHR). This polymorphism has been extensively studied since the loss of a complete exon from a gene without affecting the function of the resulting protein is very uncommon. The binding capabilities of the two GHR isoforms are considered similar [95,96,97]. The d3-GHR isoform is dominant over the fl-GHR isoform and about 50% of Europeans are hetero- or homozygous with respect to the allele encoding the d3-GHR isoform [98]. Dos Santos et al. [98] through transfection experiments demonstrated that the transduction of GH signaling through d3-GHR homo- or heterodimers was approximately 30% greater than through fl-GHR homodimers. 

Different studies have suggested that GHR polymorphism could influence GHD in adult clinical presentation and GH responsiveness. For example, vertebral fracture risk has been found decreased in adult GHD d3-GHR carriers, both in GH treated and untreated patients [99]. Glad et al. observed that adults with GHD ad fl/fl-GHR genotype showed a better IGF-I response than patients with the d3-GHR genotype, after one week of GH replacement [100]. Similar results were obtained by Moyes et al. that studied adult GHD patients after 12 months of treatment, the increase in IGF-I was significantly greater in the d3/d3 group. No difference in IGF-I increase was detected between fl/d3 and fl/fl groups and no difference in maintenance GH dose was found between different group genotypes [101]. Meyer et al. evaluated a group of adult patients with GHD derived from the prospective German Pfizer International Metabolic Study (KIMS) Pharmacogenetics Study. After one year of GH treatment, the required GH dose was lower in patients carrying one or two d3 alleles, compared with patients with the fl/fl genotype and there was no significant differences in IGF-I serum concentrations [102].

However there are other experiments with different response patterns. A group of adult GHD patients divided in two groups have been investigated after one year of GH replacement, 72 patients had fl/fl genotype, whereas 52 had at least one d3-GHR allele. Baseline values and changes in IGF-I and body fat after 12 months of GH treatment did not differ between the two genotype groups [103]. Similar results were obtained by Andujar-Plata et al. which could not find any association between the d3-GHR allele and IGF-I, the incidence of adverse events or treatment discontinuation in a group of adult-onset GHD patients [104]. In the study of Adetunji et al. the presence of the d3-GHR allele in adults with GHD did not influence the symptoms of the disease [105]. In another investigation by the same group it has been found there was no difference in the GH dose required to optimize serum IGF-I, quality of life or body composition between adult GHD carriers and non-carriers of the d3-GHR allele [106].

Another group of experiments studied the impact of the d3-GHR polymorphism on the therapeutic effect of GH replacement in longer term. Van der Klaauw et al. studied adult GHD patients (56% fl/fl, 44% d3/fl or d3/d3 GHR) after one or five years of GH replacement treatment. During one year of GH treatment, the increase in IGF-I was higher in carriers of the d3-GHR allele, compared with the fl/fl-GHR genotype. The decrease in total cholesterol (chol) and low-density lipoprotein chol was lower in the group with at least one d3-GHR allele, whereas the increase in high-density lipoprotein chol was higher, compared with non-carriers of the d3GHR allele. These differential responses of GHR genotype were no longer observed after five years of GH treatment [107]. Giavoli et al. studied prospectively adult GHD patients (48% fl/fl, 52% d3/fl or d3/d3 GHR) before and after one or five years GH replacement [96]. GH treatment normalizes IGF-I and decrease the percentage body fat both at one and five years regardless of the presence of the d3-GHR allele. At one year, an increase in high-density lipoprotein chol occurred in the d3GHR carriers compared to non-carriers. After both one and five years, the percentage of subjects with impaired glucose tolerance, that was similar in the two groups at baseline, decreased in non-carriers and doubled in d3-GHR allele carriers. In the group of d3-GHR carriers, a five year reduction in total and low-density lipoprotein chol was observed [108]. Interestingly, the d3-GHR has been found to be a marker of longevity in males, associated with increased GH sensitivity and tall stature [109].

Taking into account the studies published to date, there is little evidence that GHR polymorphism have an important impact in the clinical manifestations of the syndrome of adult GHD. The importance of the d3-GHR variant on the response of adult GHD patients to GH replacement is unclear at the present time [88,110].

## 5. Conclusions and Future Research

GH replacement exerts unquestionable beneficial effects in many patients with GHD, and its use is safe for approved indications [64]. It is necessary to identify potential candidates for treatment and confirm the GHD before the start of treatment. GH has shown beneficial effects on body composition, exercise capacity, bone structure, serum lipids, and quality of life (Table 1). The dose should be carefully individualized, to avoid side effects and adequate periodic monitoring, as is necessary (Table 2). It has not been shown that GH treatment improves overall mortality, bone fractures, clinical heart disease, or cancer.

Results of reported studies should be interpreted with caution, including those from large international databases, since all of them have methodological deficiencies, and must be assessed with carefulness and critical thinking (Table 3). Nevertheless, although our knowledge about GH replacement therapy is still imperfect, recent studies that we have commented in this review allow us to give answers based on scientific evidence to some questions of clinical relevance (Table 4).

There are ongoing preparations of prolonged-release GH, which would reduce the frequency of injections [111,112]. Nowadays, there are long-acting GH preparations in various stages of development, including depot, pegylated, and prodrug formulations, as well as non-covalent albumin-binding GH and GH-fusion protein compounds [113,114,115,116].

If its efficacy and safety are confirmed, this type of posology modification would be especially welcome in clinical practice. One of the main safety concerns of these preparations is the maintenance of supraphysiological elevations of GH or IGF-I levels, and non-physiological tissue distribution [113,115]. The possibility of modifications in the GH molecule that increase its beneficial effects and decrease its side effects is a promising way of future research. The long-term safety of GH treatment is an obligation of health professionals, the pharmaceutical industry and health authorities. Periodic monitoring of both benefits and adverse effects is necessary. Long-term studies with relevant outcome variables such as incidence of fractures, cardiovascular disease, cancer, or mortality remain necessary.

## Figures and Tables

**Table 1:** Long-term benefits and risks of growth hormone replacement therapy in adult patients with growth hormone deficiency.

Patient Data	Benefits	Risks or Drawbacks
Body composition	Reduction in fat mass Increase in lean mass Increase in muscle strength	Increase in BMI Increased waist circumference Increase of waist-hip index
Bone metabolism	Increase in bone mineral density	Effect on the incidence of fractures not clearly shown
Health-related quality of life	Improvement in quality of life questionnaires Greater benefit in patients with low quality of life at baseline	No improvement in all dimensions Probable absence of effect in patients with normal quality of life
Cardiovascular risk markers	Increase in HDL-chol Reduction of total and LDL-chol Diastolic blood pressure reduction Reduction of CRP Reduction of carotid intima-media thickness	Reduced insulin sensitivity Increase in fasting glucose and insulin Trend to the increase in the prevalence of metabolic syndrome Increase in lipoprotein (a)
Cardiovascular disease	Reduction in the incidence rate of myocardial infarction	Trend to increase in cerebrovascular disease
Neoplasms	No increase in the rate of recurrence or progression of hypothalamic-pituitary tumors No increase in overall risk of neoplasia in adults with GHD	Tendency to increase risk of second malignancy in childhood cancer survivors treated with GH in childhood There are subgroups with increased risk of certain neoplasia in adults who were treated with GH in childhood
Mortality	Tendency to decrease the global and cardiovascular mortality of hypopituitarism	Persistence of higher mortality than the general population in some studies

Abbreviations: BMI, body mass index; chol, cholesterol; HDL, high density lipoprotein; LDL, low density lipoprotein; CRP, C-reactive protein; GHD, deficiency of growth hormone.

**Table 2:** General recommendations for the treatment with GH in adults with GHD.

No.	Steps
1	Start GH replacement therapy only in adult patients with severe GHD fully demonstrated with adequate diagnostic tests
2	Use the minimum effective dose in each patient, starting with low doses
3	Use lower starting doses in the elderly and diabetics
4	Adhere the recommendations of the national and international guidelines for the follow-up of adult patients with GHD
5	Avoid IGF-I concentrations above the upper limit of normal range for the age and sex of the patient
6	Monitor blood glucose, hemoglobin A1c and lipid profile periodically and after changing doses
7	In patients with a prior history of tumors of the hypothalamus-pituitary area evaluate magnetic resonance imaging of the pituitary gland
8	Monitor the occurrence of adverse effects and inform the health authority

**Table 3:** Critical evaluation of studies on benefits and risks of GH treatment in adult patients.

No.	Steps
1	Many studies lack a control group of patients with GHD without GH treatment
2	There are no long-term, placebo-controlled studies
3	There are confounding factors that influence the results such as radiation, chemotherapy, immunosuppression, congenital diseases
4	Concomitant treatments are not always referred in the studies
5	In the long-term results we must consider the effect of age and hypopituitarism
6	In some studies follow-up is short to reach conclusive results on cardiovascular disease, neoplasia or mortality
7	Exposure to GH very variable in the patients included
8	Not all patients exposed to GH are captured in post-marketing studies
9	The studies are very heterogeneous and the designs are very disparate
10	Exposure to GH during childhood can have different effects than exposure during adult life

**Table 4:** Questions and answers about GH replacement therapy in adults with GHD.

Question	Best Available Response
Does treatment with GH improve the quality of life of patients?	Yes, in most studies
Is an improvement in body composition achieved?	Yes, it reduces fat mass and increases lean mass. In the long term too
Does treatment with GH decrease cardiovascular risk?	Yes, most cardiovascular risk markers experience a favorable change
Is the fracture rate reduced by long-term treatment?	It has not been proven
Does GH treatment modify blood glucose?	Yes, in the long term blood glucose levels rise and in some cases the incidence of glucose intolerance and diabetes increases
Does treatment during adult life increase the risk of cancer?	Probably not, but there are data that indicate that there are subgroups with higher risk of neoplasia
Does treatment with GH decrease the mortality associated with hypopituitarism?	It has not been clearly demonstrated. In adult patients treated with GH, an increase in mortality persists, in most studies

## References

[1] Diez J.J., Gomez-Pan A., Iglesias P. (1996). Towards a concept of growth hormone deficiency syndrome in adults. Med. Clin..

[2] Diez J.J. (2000). The syndrome of growth hormone deficiency in adults: Current criteria for the diagnosis and treatment. Med. Clin..

[3] Cordido F., Penalva A., Peino R., Casanueva F.F., Dieguez C. (1995). Effect of combined administration of growth hormone (GH)-releasing hormone, GH-releasing peptide-6, and pyridostigmine in normal and obese subjects. Metabolism.

[4] Cordido F., Alvarez-Castro P., Isidro M.L., Casanueva F.F., Dieguez C. (2003). Comparison between insulin tolerance test, growth hormone (GH)-releasing hormone (GHRH), GHRH plus acipimox and GHRH plus GH-releasing peptide-6 for the diagnosis of adult GH deficiency in normal subjects, obese and hypopituitary patients. Eur. J. Endocrinol..

[5] Pena-Bello L., Seoane-Pillado T., Sangiao-Alvarellos S., Outeirino-Blanco E., Varela-Rodriguez B., Juiz-Valina P., Cordido M., Cordido F. (2017). Oral glucose-stimulated growth hormone (GH) test in adult GH deficiency patients and controls: Potential utility of a novel test. Eur. J. Int. Med..

[6] Molitch M.E., Clemmons D.R., Malozowski S., Merriam G.R., Vance M.L. (2011). Evaluation and treatment of adult growth hormone deficiency: An endocrine society clinical practice guideline. J. Clin. Endocrinol. Metab..

[7] Appelman-Dijkstra N.M., Claessen K.M., Roelfsema F., Pereira A.M., Biermasz N.R. (2013). Therapy of Endocrine disease: Long-term effects of recombinant human GH replacement in adults with GH deficiency: A systematic review. Eur. J. Endocrinol..

[8] Salomon F., Cuneo R.C., Hesp R., Sonksen P.H. (1989). The effects of treatment with recombinant human growth hormone on body composition and metabolism in adults with growth hormone deficiency. N. Engl. J. Med..

[9] Elbornsson M., Gotherstrom G., Bosaeus I., Bengtsson B.A., Johannsson G., Svensson J. (2013). Fifteen years of GH replacement improves body composition and cardiovascular risk factors. Eur. J. Endocrinol..

[10] Spielhagen C., Schwahn C., Moller K., Friedrich N., Kohlmann T., Moock J., Koltowska-Haggstrom M., Nauck M., Buchfelder M., Wallaschofski H. (2011). The benefit of long-term growth hormone (GH) replacement therapy in hypopituitary adults with GH deficiency: Results of the German KIMS database. Growth Horm. IGF Res..

[11] Lamberts S.W., van den Beld A.W., van der Lely A.J. (1997). The endocrinology of aging. Science.

[12] Svensson J., Fowelin J., Landin K., Bengtsson B.A., Johansson J.O. (2002). Effects of seven years of GH-replacement therapy on insulin sensitivity in GH-deficient adults. J. Clin. Endocrinol. Metab..

[13] Filipsson Nyström H., Barbosa E.J.L., Nilsson A.G., Norrman L.-L., Ragnarsson O., Johannsson G. (2012). Discontinuing Long-Term GH Replacement Therapy—A Randomized, Placebo-Controlled Crossover Trial in Adult GH Deficiency. J. Clin. Endocrinol. Metab..

[14] Newman C.B., Carmichael J.D., Kleinberg D.L. (2015). Effects of low dose versus high dose human growth hormone on body composition and lipids in adults with GH deficiency: A meta-analysis of placebo-controlled randomized trials. Pituitary.

[15] Kargi A.Y., Merriam G.R. (2013). Diagnosis and treatment of growth hormone deficiency in adults. Nat. Rev. Endocrinol..

[16] Johannsson G., Sverrisdottir Y.B., Ellegard L., Lundberg P.A., Herlitz H. (2002). GH increases extracellular volume by stimulating sodium reabsorption in the distal nephron and preventing pressure natriuresis. J. Clin. Endocrinol. Metab..

[17] Gotherstrom G., Elbornsson M., Stibrant-Sunnerhagen K., Bengtsson B.A., Johannsson G., Svensson J. (2009). Ten years of growth hormone (GH) replacement normalizes muscle strength in GH-deficient adults. J. Clin. Endocrinol. Metab..

[18] Tritos N.A., Klibanski A. (2016). Effects of Growth Hormone on Bone. Prog. Mol. Biol. Transl. Sci..

[19] Biller B.M., Sesmilo G., Baum H.B., Hayden D., Schoenfeld D., Klibanski A. (2000). Withdrawal of long-term physiological growth hormone (GH) administration: Differential effects on bone density and body composition in men with adult-onset GH deficiency. J. Clin. Endocrinol. Metab..

[20] Underwood L.E., Attie K.M., Baptista J. (2003). Growth hormone (GH) dose-response in young adults with childhood-onset GH deficiency: A two-year, multicenter, multiple-dose, placebo-controlled study. J. Clin. Endocrinol. Metab..

[21] Allo Miguel G., Serraclara Pla A., Partida Munoz M.L., Martinez Diaz-Guerra G., Hawkins F. (2016). Seven years of follow up of trabecular bone score, bone mineral density, body composition and quality of life in adults with growth hormone deficiency treated with rhGH replacement in a single center. Ther. Adv. Endocrinol. Metab..

[22] Barake M., Klibanski A., Tritos N.A. (2014). Effects of recombinant human growth hormone therapy on bone mineral density in adults with growth hormone deficiency: A meta-analysis. J. Clin. Endocrinol. Metab..

[23] Xue P., Wang Y., Yang J., Li Y. (2013). Effects of growth hormone replacement therapy on bone mineral density in growth hormone deficient adults: A meta-analysis. Int. J. Endocrinol..

[24] Appelman-Dijkstra N.M., Claessen K.M., Hamdy N.A., Pereira A.M., Biermasz N.R. (2014). Effects of up to 15 years of recombinant human GH (rhGH) replacement on bone metabolism in adults with growth hormone deficiency (GHD): The Leiden Cohort Study. Clin. Endocrinol. (Oxf.).

[25] Gotherstrom G., Bengtsson B.A., Bosaeus I., Johannsson G., Svensson J. (2007). Ten-year GH replacement increases bone mineral density in hypopituitary patients with adult onset GH deficiency. Eur. J. Endocrinol..

[26] Rosilio M., Blum W.F., Edwards D.J., Shavrikova E.P., Valle D., Lamberts S.W., Erfurth E.M., Webb S.M., Ross R.J., Chihara K. (2004). Long-term improvement of quality of life during growth hormone (GH) replacement therapy in adults with GH deficiency, as measured by questions on life satisfaction-hypopituitarism (QLS-H). J. Clin. Endocrinol. Metab..

[27] Mo D., Blum W.F., Rosilio M., Webb S.M., Qi R., Strasburger C.J. (2014). Ten-year change in quality of life in adults on growth hormone replacement for growth hormone deficiency: An analysis of the hypopituitary control and complications study. J. Clin. Endocrinol. Metab..

[28] Elbornsson M., Horvath A., Gotherstrom G., Bengtsson B.A., Johannsson G., Svensson J. (2017). Seven years of growth hormone (GH) replacement improves quality of life in hypopituitary patients with adult-onset GH deficiency. Eur. J. Endocrinol..

[29] Giavoli C., Profka E., Verrua E., Ronchi C.L., Ferrante E., Bergamaschi S., Sala E., Malchiodi E., Lania A.G., Arosio M. (2012). GH replacement improves quality of life and metabolic parameters in cured acromegalic patients with growth hormone deficiency. J. Clin. Endocrinol. Metab..

[30] Isgaard J., Arcopinto M., Karason K., Cittadini A. (2015). GH and the cardiovascular system: An update on a topic at heart. Endocrine.

[31] Gazzaruso C., Gola M., Karamouzis I., Giubbini R., Giustina A. (2014). Cardiovascular risk in adult patients with growth hormone (GH) deficiency and following substitution with GH’An update. J. Clin. Endocrinol. Metab..

[32] Hoffman A.R., Kuntze J.E., Baptista J., Baum H.B., Baumann G.P., Biller B.M., Clark R.V., Cook D., Inzucchi S.E., Kleinberg D. (2004). Growth hormone (GH) replacement therapy in adult-onset gh deficiency: Effects on body composition in men and women in a double-blind, randomized, placebo-controlled trial. J. Clin. Endocrinol. Metab..

[33] Maison P., Griffin S., Nicoue-Beglah M., Haddad N., Balkau B., Chanson P. (2004). Impact of growth hormone (GH) treatment on cardiovascular risk factors in GH-deficient adults: A Metaanalysis of Blinded, Randomized, Placebo-Controlled Trials. J. Clin. Endocrinol. Metab..

[34] Di Somma C., Scarano E., Savastano S., Savanelli M.C., Pivonello R., Colao A. (2017). Cardiovascular alterations in adult GH deficiency. Best Pract. Res. Clin. Endocrinol. Metab..

[35] Gibney J., Wallace J.D., Spinks T., Schnorr L., Ranicar A., Cuneo R.C., Lockhart S., Burnand K.G., Salomon F., Sonksen P.H. (1999). The effects of 10 years of recombinant human growth hormone (GH) in adult GH-deficient patients. J. Clin. Endocrinol. Metab..

[36] Makimura H., Feldpausch M.N., Rope A.M., Hemphill L.C., Torriani M., Lee H., Grinspoon S.K. (2012). Metabolic Effects of a Growth Hormone-Releasing Factor in Obese Subjects with Reduced Growth Hormone Secretion: A Randomized Controlled Trial. J. Clin. Endocrinol. Metab..

[37] Fleseriu M., Hashim I.A., Karavitaki N., Melmed S., Murad M.H., Salvatori R., Samuels M.H. (2016). Hormonal Replacement in Hypopituitarism in Adults: An Endocrine Society Clinical Practice Guideline. J. Clin. Endocrinol. Metab..

[38] Kokshoorn N.E., Biermasz N.R., Roelfsema F., Smit J.W., Pereira A.M., Romijn J.A. (2011). GH replacement therapy in elderly GH-deficient patients: A systematic review. Eur. J. Endocrinol..

[39] Franco C., Johannsson G., Bengtsson B.A., Svensson J. (2006). Baseline characteristics and effects of growth hormone therapy over two years in younger and elderly adults with adult onset GH deficiency. J. Clin. Endocrinol. Metab..

[40] Morselli L.L., Nedeltcheva A., Leproult R., Spiegel K., Martino E., Legros J.J., Weiss R.E., Mockel J., Van Cauter E., Copinschi G. (2013). Impact of GH replacement therapy on sleep in adult patients with GH deficiency of pituitary origin. Eur. J. Endocrinol..

[41] Boschetti M., Agosti S., Albanese V., Casalino L., Teti C., Bezante G.P., Brunelli C., Albertelli M., Ferone D. (2017). One-year GH replacement therapy reduces early cardiac target organ damage (TOD) in adult GHD patients. Endocrine.

[42] Chikani V., Cuneo R.C., Hickman I., Ho K.K. (2016). Growth hormone (GH) enhances anaerobic capacity: Impact on physical function and quality of life in adults with GH deficiency. Clin. Endocrinol. (Oxf.).

[43] Rosen T., Bengtsson B.A. (1990). Premature mortality due to cardiovascular disease in hypopituitarism. Lancet.

[44] Svensson J., Bengtsson B.A., Rosen T., Oden A., Johannsson G. (2004). Malignant disease and cardiovascular morbidity in hypopituitary adults with or without growth hormone replacement therapy. J. Clin. Endocrinol. Metab..

[45] Tomlinson J.W., Holden N., Hills R.K., Wheatley K., Clayton R.N., Bates A.S., Sheppard M.C., Stewart P.M. (2001). Association between premature mortality and hypopituitarism. West Midlands Prospective Hypopituitary Study Group. Lancet.

[46] Claessen K.M.J.A., Appelman-Dijkstra N.M., Adoptie D.M.M.M., Roelfsema F., Smit J.W.A., Biermasz N.R., Pereira A.M. (2013). Metabolic Profile in Growth Hormone-Deficient (GHD) Adults after Long-Term Recombinant Human Growth Hormone (rhGH) Therapy. J. Clin. Endocrinol. Metab..

[47] Murray R.D., Columb B., Adams J.E., Shalet S.M. (2004). Low bone mass is an infrequent feature of the adult growth hormone deficiency syndrome in middle-age adults and the elderly. J. Clin. Endocrinol. Metab..

[48] Jorgensen A.P., Fougner K.J., Ueland T., Gudmundsen O., Burman P., Schreiner T., Bollerslev J. (2011). Favorable long-term effects of growth hormone replacement therapy on quality of life, bone metabolism, body composition and lipid levels in patients with adult-onset growth hormone deficiency. Growth Horm. IGF Res..

[49] Sommer G., Gianinazzi M.E., Kuonen R., Bohlius J., L’Allemand D., Hauschild M., Mullis P.E., Kuehni C.E. (2015). Health-Related Quality of Life of Young Adults Treated with Recombinant Human Growth Hormone during Childhood. PLoS ONE.

[50] Koltowska-Haggstrom M. (2009). Quality of life and growth hormone deficiency in adult patients in clinical evaluation and health economic assessment. Pediatr. Endocrinol. Diabetes Metab..

[51] Moock J., Albrecht C., Friedrich N., Volzke H., Nauck M., Koltowska-Haggstrom M., Kohlmann T., Wallaschofski H. (2009). Health-related quality of life and IGF-1 in GH-deficient adult patients on GH replacement therapy: Analysis of the German KIMS data and the Study of Health in Pomerania. Eur. J. Endocrinol..

[52] Urushihara H., Fukuhara S., Tai S., Morita S., Chihara K. (2007). Heterogeneity in responsiveness of perceived quality of life to body composition changes between adult- and childhood-onset Japanese hypopituitary adults with GH deficiency during GH replacement. Eur. J. Endocrinol..

[53] Wieringa G., Toogood A.A., Ryder W.D., Anderson J.M., Mackness M., Shalet S.M. (2000). Changes in lipoprotein(a) levels measured by six kit methods during growth hormone treatment of growth hormone-deficient adults. Growth Horm. IGF Res..

[54] Oliveira J.L., Aguiar-Oliveira M.H., D’Oliveira A., Pereira R.M., Oliveira C.R., Farias C.T., Barreto-Filho J.A., Anjos-Andrade F.D., Marques-Santos C., Nascimento-Junior A.C. (2007). Congenital growth hormone (GH) deficiency and atherosclerosis: Effects of GH replacement in GH-naive adults. J. Clin. Endocrinol. Metab..

[55] Cook D.M., Yuen K.C., Biller B.M., Kemp S.F., Vance M.L. (2009). American Association of Clinical Endocrinologists medical guidelines for clinical practice for growth hormone use in growth hormone-deficient adults and transition patients—2009 update. Endocr. Pract..

[56] Van Bunderen C.C., Lips P., Kramer M.H., Drent M.L. (2016). Comparison of low-normal and high-normal IGF-1 target levels during growth hormone replacement therapy: A randomized clinical trial in adult growth hormone deficiency. Eur. J. Int. Med..

[57] Florakis D., Hung V., Kaltsas G., Coyte D., Jenkins P.J., Chew S.L., Grossman A.B., Besser G.M., Monson J.P. (2000). Sustained reduction in circulating cholesterol in adult hypopituitary patients given low dose titrated growth hormone replacement therapy: A two year study. Clin. Endocrinol. (Oxf.).

[58] Ikeda H., Kudo M. (2016). Long-term follow-up results of growth hormone therapy for patients with adult growth hormone deficiency. Hormones (Athens).

[59] Attanasio A.F., Jung H., Mo D., Chanson P., Bouillon R., Ho K.K., Lamberts S.W., Clemmons D.R. (2011). Prevalence and Incidence of Diabetes Mellitus in Adult Patients on Growth Hormone Replacement for Growth Hormone Deficiency: A Surveillance Database Analysis. J. Clin. Endocrinol. Metab..

[60] Luger A., Mattsson A.F., Koltowska-Haggstrom M., Thunander M., Goth M., Verhelst J., Abs R. (2012). Incidence of diabetes mellitus and evolution of glucose parameters in growth hormone-deficient subjects during growth hormone replacement therapy: A long-term observational study. Diabetes Care.

[61] Poidvin A., Weill A., Ecosse E., Coste J., Carel J.C. (2017). Risk of Diabetes Treated in Early Adulthood after Growth Hormone Treatment of Short Stature in Childhood. J. Clin. Endocrinol. Metab..

[62] Weber M.M., Biller B.M., Pedersen B.T., Pournara E., Christiansen J.S., Hoybye C. (2017). The effect of growth hormone (GH) replacement on blood glucose homeostasis in adult nondiabetic patients with GH deficiency: Real-life data from the NordiNet((R)) International Outcome Study. Clin. Endocrinol. (Oxf.).

[63] Stochholm K., Johannsson G. (2015). Reviewing the safety of GH replacement therapy in adults. Growth Horm. IGF Res..

[64] Allen D.B., Backeljauw P., Bidlingmaier M., Biller B.M., Boguszewski M., Burman P., Butler G., Chihara K., Christiansen J., Cianfarani S. (2016). GH safety workshop position paper: A critical appraisal of recombinant human GH therapy in children and adults. Eur. J. Endocrinol..

[65] Gao Y., Katki H., Graubard B., Pollak M., Martin M., Tao Y., Schoen R.E., Church T., Hayes R.B., Greene M.H. (2012). Serum IGF1, IGF2 and IGFBP3 and risk of advanced colorectal adenoma. Int. J. Cancer.

[66] Shanmugalingam T., Bosco C., Ridley A.J., Van Hemelrijck M. (2016). Is there a role for IGF-1 in the development of second primary cancers?. Cancer Med..

[67] Dagdelen S., Cinar N., Erbas T. (2014). Increased thyroid cancer risk in acromegaly. Pituitary.

[68] Swerdlow A.J., Higgins C.D., Adlard P., Preece M.A. (2002). Risk of cancer in patients treated with human pituitary growth hormone in the UK, 1959–1985: A cohort study. Lancet.

[69] Bell J., Parker K.L., Swinford R.D., Hoffman A.R., Maneatis T., Lippe B. (2010). Long-term safety of recombinant human growth hormone in children. J. Clin. Endocrinol. Metab..

[70] Wilton P., Mattsson A.F., Darendeliler F. (2010). Growth hormone treatment in children is not associated with an increase in the incidence of cancer: Experience from KIGS (Pfizer International Growth Database). J. Pediatr..

[71] Albertsson-Wikland K., Martensson A., Savendahl L., Niklasson A., Bang P., Dahlgren J., Gustafsson J., Kristrom B., Norgren S., Pehrsson N.G. (2016). Mortality Is Not Increased in Recombinant Human Growth Hormone-treated Patients When Adjusting for Birth Characteristics. J. Clin. Endocrinol. Metab..

[72] Sklar C.A., Mertens A.C., Mitby P., Occhiogrosso G., Qin J., Heller G., Yasui Y., Robison L.L. (2002). Risk of disease recurrence and second neoplasms in survivors of childhood cancer treated with growth hormone: A report from the Childhood Cancer Survivor Study. J. Clin. Endocrinol. Metab..

[73] Ergun-Longmire B., Mertens A.C., Mitby P., Qin J., Heller G., Shi W., Yasui Y., Robison L.L., Sklar C.A. (2006). Growth hormone treatment and risk of second neoplasms in the childhood cancer survivor. J. Clin. Endocrinol. Metab..

[74] Brignardello E., Felicetti F., Castiglione A., Fortunati N., Matarazzo P., Biasin E., Sacerdote C., Ricardi U., Fagioli F., Corrias A. (2015). GH replacement therapy and second neoplasms in adult survivors of childhood cancer: A retrospective study from a single institution. J. Endocrinol. Investig..

[75] Svensson J., Bengtsson B.A. (2009). Safety aspects of GH replacement. Eur. J. Endocrinol..

[76] Carel J.-C., Ecosse E., Landier F., Meguellati-Hakkas D., Kaguelidou F., Rey G., Coste J. (2012). Long-Term Mortality after Recombinant Growth Hormone Treatment for Isolated Growth Hormone Deficiency or Childhood Short Stature: Preliminary Report of the French SAGhE Study. J. Clin. Endocrinol. Metab..

[77] Child C.J., Zimmermann A.G., Woodmansee W.W., Green D.M., Li J.J., Jung H., Erfurth E.M., Robison L.L. (2011). Assessment of primary cancers in GH-treated adult hypopituitary patients: An analysis from the Hypopituitary Control and Complications Study. Eur. J. Endocrinol..

[78] Child C.J., Conroy D., Zimmermann A.G., Woodmansee W.W., Erfurth E.M., Robison L.L. (2015). Incidence of primary cancers and intracranial tumour recurrences in GH-treated and untreated adult hypopituitary patients: Analyses from the Hypopituitary Control and Complications Study. Eur. J. Endocrinol..

[79] Olsson D.S., Trimpou P., Hallen T., Bryngelsson I.L., Andersson E., Skoglund T., Bengtsson B.A., Johannsson G., Nilsson A.G. (2017). Life expectancy in patients with pituitary adenoma receiving growth hormone replacement. Eur. J. Endocrinol..

[80] Li Z., Zhou Q., Li Y., Fu J., Huang X., Shen L. (2016). Growth hormone replacement therapy reduces risk of cancer in adult with growth hormone deficiency: A meta-analysis. Oncotarget.

[81] Pekic S., Stojanovic M., Popovic V. (2017). Controversies in the risk of neoplasia in GH deficiency. Best Pract. Res. Clin. Endocrinol. Metab..

[82] Felicetti F., Fortunati N., Arvat E., Brignardello E. (2016). GH deficiency in adult survivors of childhood cancer. Best Pract. Res. Clin. Endocrinol. Metab..

[83] van Bunderen C.C., van Nieuwpoort I.C., Arwert L.I., Heymans M.W., Franken A.A.M., Koppeschaar H.P.F., van der Lely A.J., Drent M.L. (2011). Does Growth Hormone Replacement Therapy Reduce Mortality in Adults with Growth Hormone Deficiency? Data from the Dutch National Registry of Growth Hormone Treatment in Adults. J. Clin. Endocrinol. Metab..

[84] Gaillard R.C., Mattsson A.F., Akerblad A.C., Bengtsson B.A., Cara J., Feldt-Rasmussen U., Koltowska-Haggstrom M., Monson J.P., Saller B., Wilton P. (2012). Overall and cause-specific mortality in GH-deficient adults on GH replacement. Eur. J. Endocrinol..

[85] Quigley C.A., Child C.J., Zimmermann A.G., Rosenfeld R.G., Robison L.L., Blum W.F. (2017). Mortality in Children Receiving Growth Hormone Treatment of Growth Disorders: Data From the Genetics and Neuroendocrinology of Short Stature International Study. J. Clin. Endocrinol. Metab..

[86] Waters M.J. (2016). The growth hormone receptor. Growth Horm. IGF Res..

[87] Bergan-Roller H.E., Sheridan M.A. (2018). The growth hormone signaling system: Insights into coordinating the anabolic and catabolic actions of growth hormone. Gen. Comp. Endocrinol..

[88] Boguszewski C.L., Barbosa E.J.L., Svensson P.A., Johannsson G., Glad C.A.M. (2017). MECHANISMS IN ENDOCRINOLOGY: Clinical and pharmacogenetic aspects of the growth hormone receptor polymorphism. Eur. J. Endocrinol..

[89] Leung D.W., Spencer S.A., Cachianes G., Hammonds R.G., Collins C., Henzel W.J., Barnard R., Waters M.J., Wood W.I. (1987). Growth hormone receptor and serum binding protein: Purification, cloning and expression. Nature.

[90] Brooks A.J., Dai W., O’Mara M.L., Abankwa D., Chhabra Y., Pelekanos R.A., Gardon O., Tunny K.A., Blucher K.M., Morton C.J. (2014). Mechanism of activation of protein kinase JAK2 by the growth hormone receptor. Science.

[91] Sawada T., Arai D., Jing X., Miyajima M., Frank S.J., Sakaguchi K. (2017). Molecular interactions of EphA4, growth hormone receptor, Janus kinase 2, and signal transducer and activator of transcription 5B. PLoS ONE.

[92] Soendergaard C., Young J.A., Kopchick J.J. (2017). Growth Hormone Resistance-Special Focus on Inflammatory Bowel Disease. Int. J. Mol. Sci..

[93] Barton D.E., Foellmer B.E., Wood W.I., Francke U. (1989). Chromosome mapping of the growth hormone receptor gene in man and mouse. Cytogenet. Cell Genet..

[94] Godowski P.J., Leung D.W., Meacham L.R., Galgani J.P., Hellmiss R., Keret R., Rotwein P.S., Parks J.S., Laron Z., Wood W.I. (1989). Characterization of the human growth hormone receptor gene and demonstration of a partial gene deletion in two patients with Laron-type dwarfism. Proc. Natl. Acad. Sci. USA.

[95] Sobrier M.L., Duquesnoy P., Duriez B., Amselem S., Goossens M. (1993). Expression and binding properties of two isoforms of the human growth hormone receptor. FEBS Lett..

[96] Bass S.H., Mulkerrin M.G., Wells J.A. (1991). A systematic mutational analysis of hormone-binding determinants in the human growth hormone receptor. Proc. Natl. Acad. Sci. USA.

[97] Urbanek M., Russell J.E., Cooke N.E., Liebhaber S.A. (1993). Functional characterization of the alternatively spliced, placental human growth hormone receptor. J. Biol. Chem..

[98] Dos Santos C., Essioux L., Teinturier C., Tauber M., Goffin V., Bougneres P. (2004). A common polymorphism of the growth hormone receptor is associated with increased responsiveness to growth hormone. Nat. Genet..

[99] Mormando M., Chiloiro S., Bianchi A., Giampietro A., Angelini F., Tartaglione L., Nasto L., Milardi D., Formenti A.M., Giustina A. (2016). Growth hormone receptor isoforms and fracture risk in adult-onset growth hormone-deficient patients. Clin. Endocrinol. (Oxf.).

[100] Glad C.A., Barbosa E.J., Filipsson Nystrom H., Carlsson L.M., Nilsson S., Nilsson A.G., Svensson P.A., Johannsson G. (2014). SNPs within the GH-signaling pathway are associated with the early IGF1 response to GH replacement therapy in GHD adults. Eur. J. Endocrinol..

[101] Moyes V.J., Walker D.M., Owusu-Antwi S., Maher K.T., Metherell L., Akker S.A., Monson J.P., Clark A.J., Drake W.M. (2010). d3-GHR genotype does not explain heterogeneity in GH responsiveness in hypopituitary adults. Clin. Endocrinol. (Oxf.).

[102] Meyer S., Schaefer S., Stolk L., Arp P., Uitterlinden A.G., Plockinger U., Stalla G.K., Tuschy U., Weber M.M., Weise A. (2009). Association of the exon 3 deleted/full-length GHR polymorphism with recombinant growth hormone dose in growth hormone-deficient adults. Pharmacogenomics.

[103] Barbosa E.J., Palming J., Glad C.A., Filipsson H., Koranyi J., Bengtsson B.A., Carlsson L.M., Boguszewski C.L., Johannsson G. (2009). Influence of the exon 3-deleted/full-length growth hormone (GH) receptor polymorphism on the response to GH replacement therapy in adults with severe GH deficiency. J. Clin. Endocrinol. Metab..

[104] Andujar-Plata P., Fernandez-Rodriguez E., Quinteiro C., Casanueva F.F., Bernabeu I. (2015). Influence of the exon 3 deletion of GH receptor and IGF-I level at diagnosis on the efficacy and safety of treatment with somatotropin in adults with GH deficiency. Pituitary.

[105] Adetunji O.R., Blair J.C., Javadpour M., Alfirevic A., Pirmohamed M., MacFarlane I.A. (2010). Deletion of exon 3 in the growth hormone receptor gene in adults with growth hormone deficiency: Comparison of symptomatic and asymptomatic patients. Clin. Endocrinol. (Oxf.).

[106] Adetunji O.R., MacFarlane I.A., Javadpour M., Alfirevic A., Pirmohamed M., Blair J.C. (2009). The d3/fl-GH receptor gene polymorphism does not influence quality of life and body composition in GH-deficient adults receiving GH replacement therapy. Eur. J. Endocrinol..

[107] Van der Klaauw A.A., van der Straaten T., Baak-Pablo R., Biermasz N.R., Guchelaar H.J., Pereira A.M., Smit J.W., Romijn J.A. (2008). Influence of the d3-growth hormone (GH) receptor isoform on short-term and long-term treatment response to GH replacement in GH-deficient adults. J. Clin. Endocrinol. Metab..

[108] Giavoli C., Ferrante E., Profka E., Olgiati L., Bergamaschi S., Ronchi C.L., Verrua E., Filopanti M., Passeri E., Montefusco L. (2010). Influence of the d3GH receptor polymorphism on the metabolic and biochemical phenotype of GH-deficient adults at baseline and during short- and long-term recombinant human GH replacement therapy. Eur. J. Endocrinol..

[109] Ben-Avraham D., Govindaraju D.R., Budagov T., Fradin D., Durda P., Liu B., Ott S., Gutman D., Sharvit L., Kaplan R. (2017). The GH receptor exon 3 deletion is a marker of male-specific exceptional longevity associated with increased GH sensitivity and taller stature. Sci. Adv..

[110] Boguszewski C.L. (2017). Update on GH therapy in adults. F1000Research.

[111] Biller B.M., Ji H.J., Ahn H., Savoy C., Siepl E.C., Popovic V., Coculescu M., Roemmler J., Gavrila C., Cook D.M. (2011). Effects of once-weekly sustained-release growth hormone: A double-blind, placebo-controlled study in adult growth hormone deficiency. J. Clin. Endocrinol. Metab..

[112] Kramer W.G., Jaron-Mendelson M., Koren R., Hershkovitz O., Hart G. (2017). Pharmacokinetics, Pharmacodynamics, and Safety of a Long-Acting Human Growth Hormone (MOD-4023) in Healthy Japanese and Caucasian Adults. Clin. Pharmacol. Drug Dev..

[113] Hoybye C., Cohen P., Hoffman A.R., Ross R., Biller B.M., Christiansen J.S. (2015). Status of long-acting-growth hormone preparations—2015. Growth Horm. IGF Res..

[114] Hoybye C., Pfeiffer A.F., Ferone D., Christiansen J.S., Gilfoyle D., Christoffersen E.D., Mortensen E., Leff J.A., Beckert M. (2017). A phase 2 trial of long-acting TransCon growth hormone in adult GH deficiency. Endocr. Connect..

[115] Christiansen J.S., Backeljauw P.F., Bidlingmaier M., Biller B.M., Boguszewski M.C., Casanueva F.F., Chanson P., Chatelain P., Choong C.S., Clemmons D.R. (2016). Growth Hormone Research Society perspective on the development of long-acting growth hormone preparations. Eur. J. Endocrinol..

[116] Sprogoe K., Mortensen E., Karpf D.B., Leff J.A. (2017). The rationale and design of TransCon Growth Hormone for the treatment of growth hormone deficiency. Endocr. Connect..

